# Comparative genome analysis of multidrug-resistant *Pseudomonas aeruginosa* JNQH-PA57, a clinically isolated mucoid strain with comprehensive carbapenem resistance mechanisms

**DOI:** 10.1186/s12866-021-02203-4

**Published:** 2021-05-01

**Authors:** Mingju Hao, Wanshan Ma, Xiutao Dong, Xiaofeng Li, Fang Cheng, Yujiao Wang

**Affiliations:** 1 Department of Clinical Laboratory Medicine, The First Affiliated Hospital of Shandong First Medical University & Shandong Provincial Qianfoshan Hospital, Jinan, Shandong China; 2 Shandong Medicine and Health Key Laboratory of Laboratory Medicine, Jinan, Shandong China

**Keywords:** Complete genome sequencing, *Pseudomosa aeruginosa*, Mucoid strain, Alginates-overproducing, Carbapenem resistance mechanisms

## Abstract

**Background:**

The prevalence of clinical multidrug-resistant (MDR) *Pseudomonas aeruginosa* has been increasing rapidly worldwide over the years and responsible for a wide range of acute and chronic infections with high mortalities. Although hundreds of complete genomes of clinical *P. aeruginosa* isolates have been sequenced, only a few complete genomes of mucoid strains are available, limiting a comprehensive understanding of this important group of opportunistic pathogens. Herein, the complete genome of a clinically isolated mucoid strain *P. aeruginosa* JNQH-PA57 was sequenced and assembled using Illumina and Oxford nanopore sequencing technologies. Genomic features, phylogenetic relationships, and comparative genomics of this pathogen were comprehensively analyzed using various bioinformatics tools. A series of phenotypic and molecular-genetic tests were conducted to investigate the mechanisms of carbapenem resistance in this strain.

**Results:**

Several genomic features of MDR *P. aeruginosa* JNQH-PA57 were identified based on the whole-genome sequencing. We found that the accessory genome of JNQH-PA57 including several prophages, genomic islands, as well as a PAPI-1 family integrative and conjugative element (ICE), mainly contributed to the larger genome of this strain (6,747,067 bp) compared to other popular *P. aeruginosa* strains (with an average genome size of 6,445,223 bp) listed in *Pseudomonas *Genome Database. Colony morphology analysis and biofilm crystal staining assay respectively demonstrated an enhanced alginate production and a thicker biofilm formation capability of JNQH-PA57. A deleted mutation at nt 424 presented in *mucA* gene, resulted in the upregulated expression of a sigma-factor AlgU and a GDP mannose dehydrogenase AlgD, which might explain the mucoid phenotype of this strain. As for the carbapenem resistance mechanisms, our results revealed that the interplay between impaired OprD porin, chromosomal β-lactamase OXA-488 expression, MexAB-OprM and MexXY-OprM efflux pumps overexpression, synergistically with the alginates-overproducing protective biofilm, conferred the high carbapenem resistance to *P. aeruginosa* JNQH-PA57.

**Conclusion:**

Based on the genome analysis, we could demonstrate that the upregulated expression of *algU* and *algD*, which due to the truncation variant of MucA, might account for the mucoid phenotype of JNQH-PA57. Moreover, the resistance to carbapenem in *P. aeruginosa* JNQH-PA57 is multifactorial. The dataset presented in this study provided an essential genetic basis for the comprehensive cognition of the physiology, pathogenicity, and carbapenem resistance mechanisms of this clinical mucoid strain.

**Supplementary Information:**

The online version contains supplementary material available at 10.1186/s12866-021-02203-4.

## Background

*Pseudomonas aeruginosa* is an opportunistic pathogen that can cause a wide range of acute and chronic infections. It is also responsible for the life-threatening infections in patients with malignancy, immunosuppression, burns, and those who performed mechanical ventilation [1]. *P. aeruginosa* possesses versatile abilities to evade host immune response and cause stubborn persistent infections, such as switching to mucoid phenotype, reducing its motility and virulence, presenting a perpetual biofilm state, and developing strong resistance [2, 3]. Even worse, researches showed that the prevalence of clinical multidrug-resistant (MDR) strains has been increasing rapidly worldwide over the years [4–6].

The emergence of carbapenem-resistant *P. aeruginosa* strains greatly compromised the effectiveness of first-line carbapenems treatment. In addition to the acquisition of carbapenem-hydrolyzing β-lactamases [7], carbapenem resistance in *P. aeruginsa* is known to be the result of interplay between the reduction of permeability, overexpression of the efflux pumps, as well as activity of an inducible chromosomal β-lactamase AmpC [8]. Furthermore, *P. aeruginosa* is also notorious for the hallmark of conversion to the biofilm-growing mucoid (alginate-producing) phenotype, which makes this pathogen adaptable for long-term persistence in chronic infections [9]. Once the chronic infection is established, *P. aeruginosa* may acquire mutations leading to the conversion of mucoid phenotype, and exhibit reinforced recalcitrance to clearance by the immune system and antimicrobial therapy [9].

The genome size of *P. aeruginosa* commonly varies from 5.5 Mb to 7 Mb [10, 11]. The incredible metabolic versatility and strong antibiotics resistance of *P. aeruginosa* are generally attributed to genomic diversity [11]. The accessory genomes are usually composed of horizontally transferable elements which include integrative and conjugative elements (ICEs), genomic islands (GIs), prophages, transposons, insertion sequences (ISs), and integrons [12], and they often contribute to the unique physiology, pathogenesis and antibiotics resistances of the corresponding strains [7, 13]. Although over hundreds of complete genomes of clinical *P. aeruginosa* isolates were deposited in the National Center for Biotechnology Information (NCBI) GenBank, only a few complete genomes of mucoid strains are available (including but not limited to *P. aeruginosa* FRD1 (NZ_CP010555.1) [14], *P. aeruginosa* DK1-NH57388A (NZ_LN870292.1) [15]), limiting a comprehensive understanding of this important group of opportunistic pathogens. A better understanding of the genetic diversity of mucoid *P. aeruginosa* as well as its antibiotic resistance mechanisms is critical to achieving more targeted therapy of chronic lung infections.

The main goal of this study was to determine and analyse the complete genomic sequence of an MDR *P. aeruginosa* strain with mucoid phenotype isolated from respiratory sputum. We sought genomic and phenotypic differences between this mucoid strain and three widely studied reference strains, *P. aeruginoas* PAO1, PA14 and ATCC 27853. The genomic features of strain JNQH-PA57 were compared with those of three reference strains, and the phenotypic characterizations of colony morphology and biofilm formation of these four strains were performed in vitro. Moreover, the expression levels of *algU* and *algD*, which related to alginate biosynthesis in JNQH-PA57 and PAO1, were assessed as well. Finally, we conducted a series of phenotypic and molecular-genetic tests to investigate the mechanisms of carbapenem resistance in *P. aeruginosa* JNQH-PA57. This work represents the detailed genomic comparison of a clinically isolated mucoid *P. aeruginosa* carrying comprehensive multidrug-resistance mechanisms.

## Results

### Extended resistance spectrum of mucoid strain *P. aeruginosa* JNQH-PA57

The *P. aeruginosa* mucoid strain JNQH-PA57 was isolated from a sputum sample of an 86-year-old male patient hospitalized with symptoms of heart failure and pneumonia. This strain was isolated as the causative pathogen of pneumonia which was highly resistant to piperacillin, ceftazidime, piperacillin/tazobactam, imipenem, meropenem, aztreonam. This strain was also resistant to cefepime, ticarcillin/clavulanic acid; but susceptible to amikacin, tobramycin, levofloxacin, ciprofloxacin, and colistin (Table 1).

**Table 1:** Minimal inhibitory concentrations (MICs) of some antibiotics for mucoid strain *P. aeruginosa* JNQH-PA57

Category	Antibiotics	MIC μg/ml	Resistance
Penicillins	Piperacillin	> 256	R
Cephalosporins	Ceftazidime	> 128	R
	Cefepime	> 32	R
Carbapenems	Imipenem	> 16	R
	Meropenem	> 32	R
Monobactams	Aztreonam	> 128	R
Penicillins+β-Lactamase inhibitor	Piperacillin/Tazobactam	> 256	R
	Ticarcillin/Clavulanic Acid	> 128	R
Aminoglycosides	Amikacin	< 2	S
	Tobramycin	< 1	S
Fluoroquinolones	Levofloxacin	< 1	S
	Ciprofloxacin	< 0.25	S
Polymyxins	Colistin	< 0.5	S

**Fig. 1:**
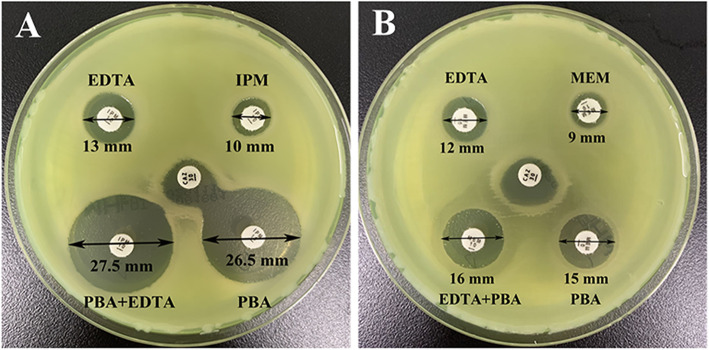
Identification of the carbapenemase production with three combined-disc tests. **a** Combined discs consisting of imipenem alone, imipenem with PBA or EDTA respectively, or imipenem with PBA plus EDTA. **b** Combined discs consisting of meropenem alone, meropenem with PBA or EDTA respectively, or meropenem with PBA plus EDTA. Augmentation of the inhibition zone by ≥5 mm was considered as a positive combined-disc test result

Detailed test results of the phenotypic screening for carbapenem resistance were shown in Fig. 1. *P. aeruginosa* JNQH-PA57 showed a ≥ 5 mm enlargement in the inhibition zone diameters of the combined discs with PBA or EDTA plus PBA, compared with imipenem or meropenem alone, whereas the combined-disc that used imipenem/meropenem with or without EDTA showed an unapparent difference in the inhibition zone diameters (Fig. 1). These results suggested that JNQH-PA57 was negative for metallo-β-lactamase production but positive for serine β-lactamase production.

### Genome features and phylogenetic relationship of *P. aeruginosa* JNQH-PA57

The hybrid genome assembly resulted in a single circular chromosome of 6,747,067 bp with an average GC content of 66.03%, which is larger than other published complete genomes of popular *P. aeruginosa* strains (with an average genome size of 6,477,604 bp) in *Pseudomonas* Genome Database (PGDB) (Additional file 1: Table S1). A total of 6239 genes were predicted from JNQH-PA57 genome by NCBI Prokaryotic Genome Annotation Pipeline (PGAP) server, which included 6087 coding sequences (CDS) and identified 63 tRNA genes and 12 rRNA operons and 4 ncRNA genes (Table S1).

**Fig. 2:**
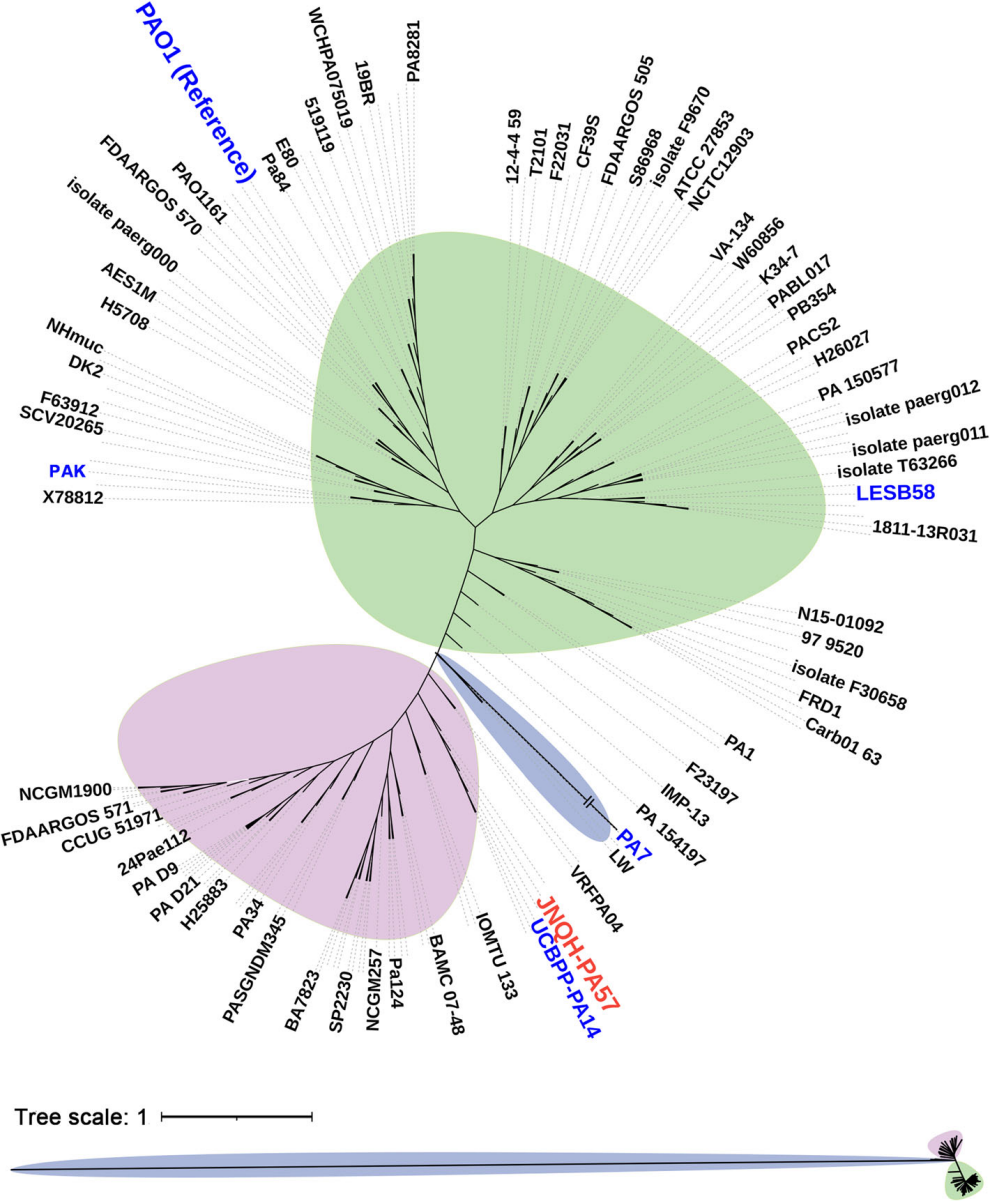
Unrooted phylogenomic tree of clinically isolated 158 *P. aeruginosa* (including the JNQH-PA57)*.* The phylogenomic tree was constructed based on identifying SNPs between the reference PAO1 genome and other 157 clinically isolated *P. aeruginosa* genomes. Strains are clustered into three groups (group 1: green, group 2: pink and group 3: blue). Original appearance of this tree was present at the bottom in which the scale bars represent the number of substitutions per site

In order to examine the levels of genetic diversities and phylogenetic relationships between JNQH-PA57 and publicly available *P. aeruginosa* genome, a total of 157 complete genomes of clinical isolates *P. aeruginosa* including PAO1 were downloaded from the NCBI database. These 157 strains were listed in PGDB [16] as a complete genome represent global *P. aeruginosa* clinical collections (PGDB version 20.2, latest update on September 21, 2020). SNP genome alignment showed that a total of 5,020,270 SNP sites were identified amongst 158 isolates (including JNQH-PA57) (Fig. 2, Additional file 2: Table S2). Phylogenetic tree revealed all strains can be broadly clustered into two major groups with a minor branch containing three *P.aeruginosa* strains (including a multi-resistant taxonomic outlier PA7) [17] as a third group (Fig. 2). Group 1 is the larger one which includes the two widely studied reference strains PAO1 [18] and ATCC 27853 [19], as well as some notable clinical strains such as LESB58 [20], DK2 [21] and PAK [22]. Group 2 is a smaller one which contains the well-studied highly virulent strain PA14 which has been used extensively to study the contribution of putative virulence factors to disease [23]. It is apparent that *P. aeruginosa* JNQH-PA57 is very closely related to PA14, and both are clustered into the sub-lineage within group 2.

### Comparison of *P. aeruginosa* JNQH-PA57 genome with the PAO1, PA14 and ATCC 27853 genomes

**Fig. 3:**
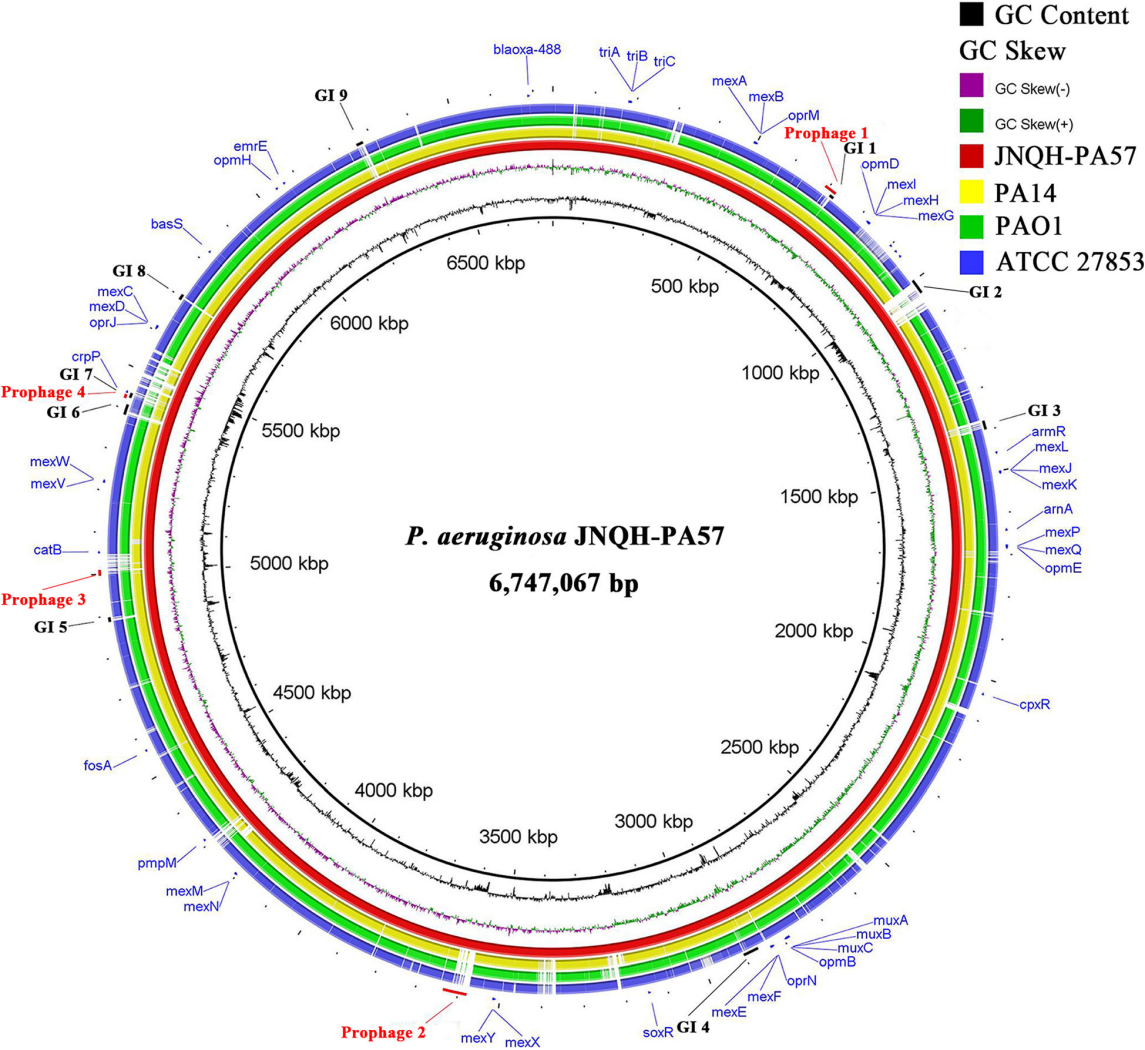
Circular genome map of *P. aeruginosa* JNQH-PA57 compared to ATCC 27853, PAO1 and PA14. Map and underlying analysis were performed with the BLAST Ring Image Generator (BRIG) v0.95. Rings from the outside inward: circle 1, ATCC 27853 genome; circle 2, PAO1 genome; circle 3, PA14 genome; circle 4, JNQH-PA57 genome; circle 5, GC skew; circle 6, GC content. GIs and prophages, predicted by IslandViewer and prophages respectively, were indicated on the outside of the map. A total of 47 AMR genes were curated from the Comprehensive Antibiotic Resistance Database (CARD) and indicated on the map

**Fig. 4:**
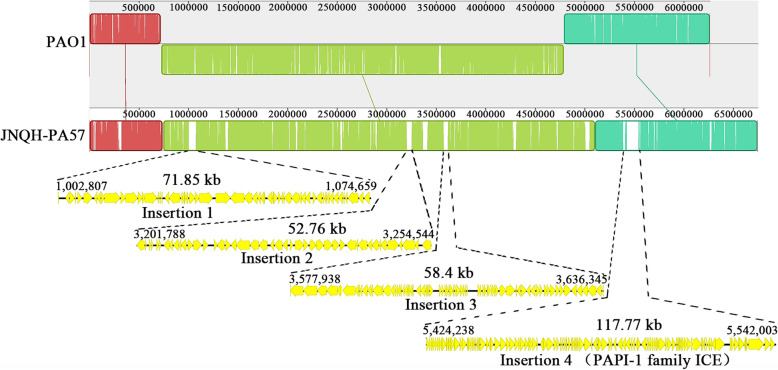
Mauve alignment of JNQH-PA57 and PAO1 revealed major structural variations between the genomes of these two strains. Blocks indicated regions with percentage of nucleotide sequence identity higher than 75%. The inversion between *rrnA* and *rrnB* rRNA operons was colored in aqua. Bottom panel indicated positions and schematic gene organization of four large insertion regions (> 50 kb) which were absent in PAO1 genome

To identify the strain-specific genome features in the JNQH-PA57 genome, the complete genome of JNQH-PA57 was further compared with those of three widely studied reference *P. aeruginosa* strains, PAO1 [18], PA14 [24] and ATCC 27853 [19]. Comparative analysis showed that the genome of JNQH-PA57 is larger than that of PA14, PAO1 and ATCC 27853. Several relatively large regions, which consisted of several prophages and GIs, mainly conduce to the large genome of JNQH-PA57 (visible in Fig. 3). The genome of JNQH-PA57 shared 676,422 identical sites (86.7%) with that of PAO1. Even though, the architecture of the JNQH-PA57 genome contains several major differences (Fig. 4). Firstly, the large inversion between ribosomal RNA operons *rrnA* and *rrnB* observed in PAO1 [18] does not exist in the genome of *P. aeruginosa* JNQH-PA57 (Fig. 4). Secondly, the latter possesses several large insertion regions (> 50 kb) which is absent in PAO1 genome (Fig. 4 and Additional file 3: Table S3). The first insertion is between PA4040 (DUF4340 domain-containing protein) and PA4041 (mandelate racemase), with an integrase in 3′-end of the sequence, is composed of a 71.85 kb sequence with a coding capacity for 62 open reading frames (ORFs). Additionally, JNQH-PA57 lacks a 4150 bp fragment containing PA2218, PA2219, and PA2210 genes and has a 52,757 bp insertion downstream of PA2221, which is the second insertion identified in JNQH-PA57 genome. The third insertion between PA1965 and PA1964 (located between the 3,577,938 bp and 3,636,345 bp in JNQH-PA57 genome), which is composed of a 58.4 kb fragment with a coding capacity for 77 ORFs, is identified as a prophage specifically apparent in the genome of JNQH-PA57, which does not exist in the genomes of PAO1, PA14 and ATCC 27853. Further aligning this insertion with the NCBI database revealed that this fragment existed in the JNQH-PA57 uniquely. The fourth, as well as the largest insertion region, is a 117,766 bp fragment located between PA4541 (*tpsA4*) and PA4542 (*clpB*), flanked by 46 bp identical *attL* and *attR* sequences. This insertion fragment displays a significant similarity to PAPI-1 family ICEs (Fig. 4).

### GIs and prophages identified in the genome of JNQH-PA57

We predicted the GIs in the JNQH-PA57 genome with the Island Viewer 4 server [25]. A total of nine GIs were identified in the genome of JNQH-PA57 by the Island Viewer 4 server (Table 2). The lengths of these GIs range from 11,253 bp to 37,691 bp. Most of the genes located in the genomic islands encode integrases, transposases (including IS110, IS3 and IS4 family transposases), virulence factors (such as the two-partner secretion system exoprotein TpsA1, type-IV pilus biogenesis and extracellular protein secretion related proteins PilB, PilD, and type III secretion system-related proteins FliR, FliQ, FliP) (Table 2 and Additional file 4: Table S4), which may be important for the survival, nosocomial spread and pathogenicity of the JNQH-PA57.

**Table 2:** List of GIs identified in *P. aeruginosa* JNQH-PA57

ID	Start	End	Size (bp)	Gene locus tag rang	Genes of interest
GI 1	713,078	724,317	11,239	H5409_03350- H5409_03400	Prophage 1, anthranilate phosphoribosyltransferase TrpC, indole-3-glycerol phosphate synthase TrpD
GI 2	998,362	1,034,278	35,916	H5409_04670- H5409_04810	Hypothetical proteins, IS110 family transposase
GI 3	1,376,175	1,397,681	21,506	H5409_06435- H5409_06535	VapC family toxin, conjugative transfer protein TrbJ, TrbL
GI 4	2,864,029	2,901,720	37,691	H5409_13445- H5409_13535	Proteins involved in iron acquisition and virulence, IS4 family transposase, IS3 family transposase
GI 5	4,885,149	4,896,402	11,253	H5409_22590- H5409_22650	tyrosine-type recombinase/integrase, adenylyltransferase
GI 6	5,386,356	5,411,738	25,382	H5409_24840- H5409_24980	site-specific integrase, pilin, pilus assembly ATPase PilB, prepilin peptidase PilD
GI 7	5,428,676	5,440,617	11,941	H5409_25065- H5409_25160	Integrative conjugative elements
GI 8	5,692,873	5,707,176	14,303	H5409_26330- H5409_26415	50S ribosomal protein, IS3 family transposase, type II toxin-antitoxin system RelE/ParE family toxin
GI 9	6,261,122	6,277,771	16,649	H5409_28890- H5409_29015	Type III secretion system related proteins FliR, FliQ, FliP

In addition, four prophages in the genome of *P. aeruginosa* JNQH-PA57, designated as Prophage 1–4, have been predicted using PHASTER [26] (Fig. 3 and Table 3). Prophage 1 is a 30,651 bp fragment located between H5409_03200 and H5409_03390. This prophage is highly conserved in all available *P. aeruginosa* genomes, based on the PHASTER database [26]. Prophage 2 is the largest prophage in this strain with a size of 58,408 bp, in which most ORFs encode phage-related proteins such as phage head and tail, transposases and integrases (Additional file 5: Table S5). Prophage 3 is a 13,578 bp fragment located between H5409_23135 and H5409_23230, which shares high similarity with *Pseudomonas* phage Pf1, a filamentous bacteriophage identified by Hill et al. [27]. The last prophage is regarded as an incomplete prophage with a very low predicted score, which contains only ten phage hit proteins.

**Table 3:** List of prophages identified in *P. aeruginosa* JNQH-PA57

Prophage ID	Region Length	Completeness	Score	Total Proteins	Region Position	Close Phage	GC %
Prophage 1	30.6 kb	intact	150	37	691,434–722,084	*Pseudomonas* phage phiCTX	64.15%
Prophage 2	57 kb	intact	120	58	3,579,267–3,636,345	*Pseudomonas* phage vB_PaeP_Tr60_Ab3	61.29%
Prophage 3	13.5 kb	intact	96	17	4,996,552–5,010,118	*Pseudomonas* phage Pf1	56.24%
Prophage 4	9.4 kb	incomplete	10	16	5,424,375–5,433,867	Bacill_phBC6A51	61.57%

### JNQH-PA57 genome contains a PAPI-1 family ICE

**Fig. 5:**
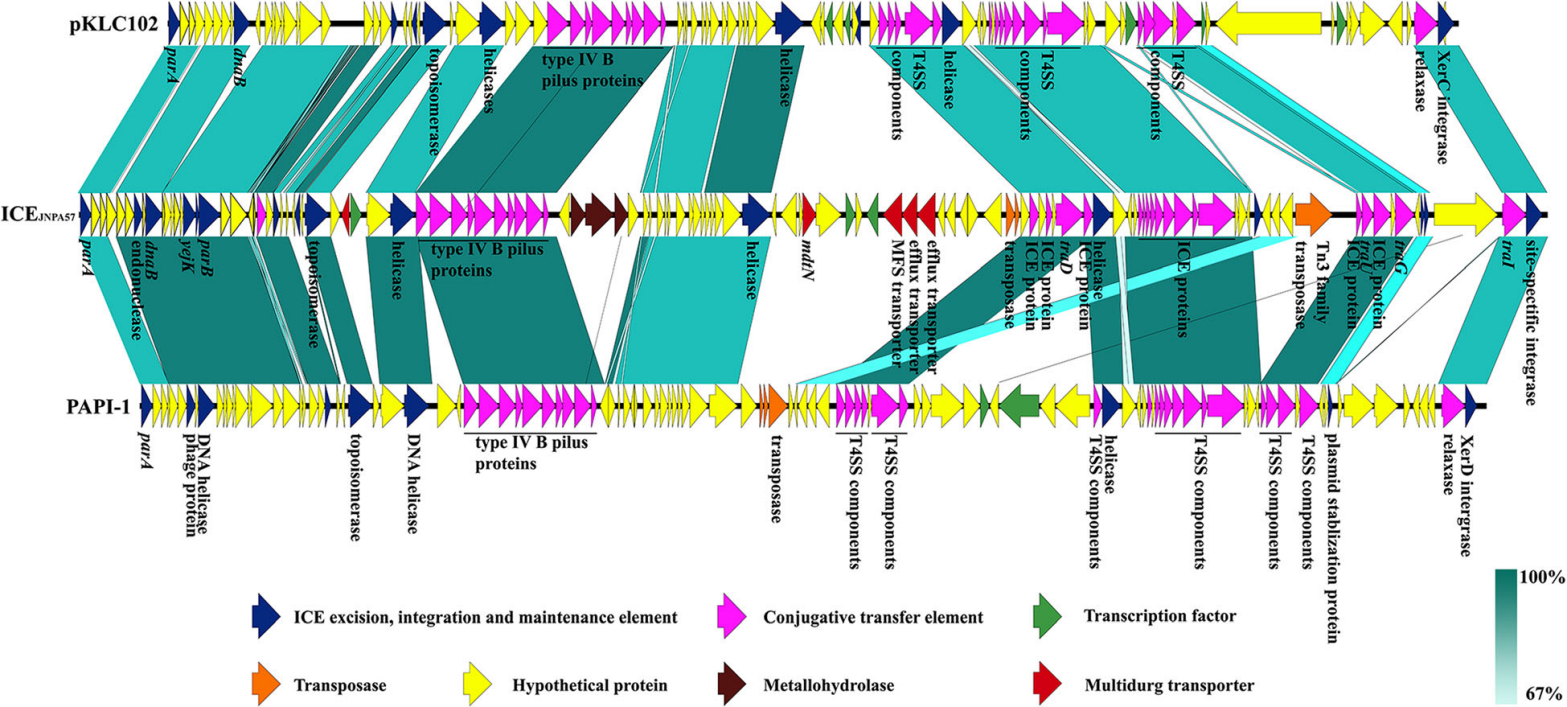
Comparative genomics of ICE_JNPA57_ and other two PAPI-1 family ICEs. The genes in these three ICEs were exhibited in arrows with different colors to note on their functional classes. The length of each arrow represented a proportion of the gene size, and the direction of the arrow indicated the gene transcription direction. The shades among these three ICE represented the sequence identity of linked regions

Based on the sequence alignment and ICEberg database [28], a 117,766 bp putative ICE, with the type IV secretion system (T4SS), is located on the chromosome (between the position 5,424,238 bp to 5,542,003 bp) of this strain (Fig. 4) and bracketed by two 46 bp highly conserved direct repeats (5′-ATGGTGGGTCGTGTAGGATTCGAACCTACGACCAATTGGTTAAAAG-3′), named *attL* and *attR*, on both sides. This element, named ICE_JNPA57_, which is located adjacent to a tRNA cluster integration site, starts with a gene encoding ParA family protein and ends with a site-specific recombinase (Fig. 5). The sequence and architecture of operons flanking the putative integration site in this putative ICE showed significant similarities with those in the PAPI-1 family ICEs [13], especially with pathogenicity islands pKLC102 in *P. aeruginosa* clone C strains (with 61% coverage and 96.14% identity) [29] and the pathogenicity island PAPI-1 in *P. aeruginosa* PA14 (with 63% coverage and 97.95% identity) [30] (Fig. 5). Comparison between these three PAPI-1 family ICEs revealed that the conjugation machinery elements which contain the T4SS, as well as genes those encoding putative ICE family proteins, relaxases and site-directed integrase, were highly conserved in these three ICEs, especially between the ICE_JNPA57_ in JNQH-PA57 and PAPI-1 in PA14 (Fig. 5). In addition, 121 ORFs were predicted in ICE_JNPA57_, of which at least 30% were “hypothetical proteins” (Additional file 6: Table S6). It was noted that two segments, which contained several cargo genes encoding the putative metallohydrolases and multidrug transporters respectively, were exclusively existed in the ICE_JNPA57_ (Fig. 5).

### Clusters of orthologous groups comparison

**Fig. 6:**
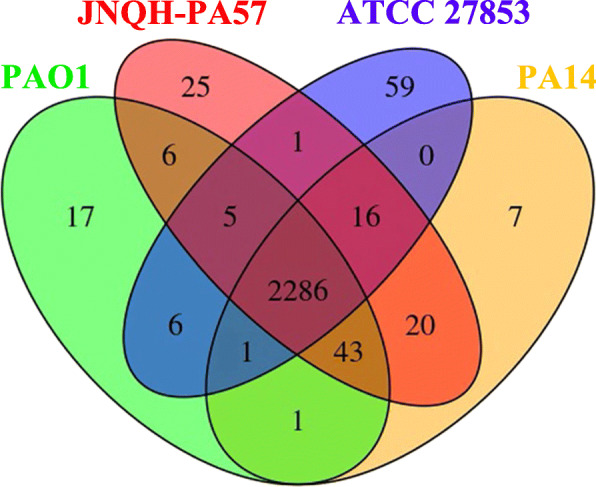
COG comparisons of *P. aeruginosa* strains. Venn diagram showed the number of exclusive and shared genes among four *P. aeruginosa* strains: PAO1, JNQH-PA57, ATCC 27853, and PA14. The number within the ellipses in the venn diagram represent the number of unique genes, those shared among two, three and all four strains of PAO1, JNQH-PA57, ATCC 27853, PA14 strains were based on the COG gene annotations

Comparison of Clusters of Orthologous Groups (COG) annotations revealed a total of 25 COGs are exclusively present in *P. aeruginosa* JNQH-PA57 compared with PAO1, PA14 and ATCC 27853 (Fig. 6 and Additional file 7: Table S7). Most of these COGs are efflux transporter related proteins, which might contribute to the extended resistance spectrum exhibited by this strain. The details of the COG communities’ distribution among these four strains were exhibited in Fig. 6. Of note, JNQH-PA57 shares a total of 2365 COGs with PA14 (Fig. 6), a much higher number than that with PAO1 (shares 2340 COGs) and ATCC 27853 (shares 2308 COGs). These shared COGs also echoed the phylogenetic relationship as described above, that JNQH-PA57 has a rather closer genetic relationship with PA14 than with PAO1 and ATCC 27853.

### *P. aeruginosa* JNQH-PA57 exhibits distinctive biofilm-growing mucoid (alginate-producing) colony morphology due to the derepression of AlgU

**Fig. 7:**
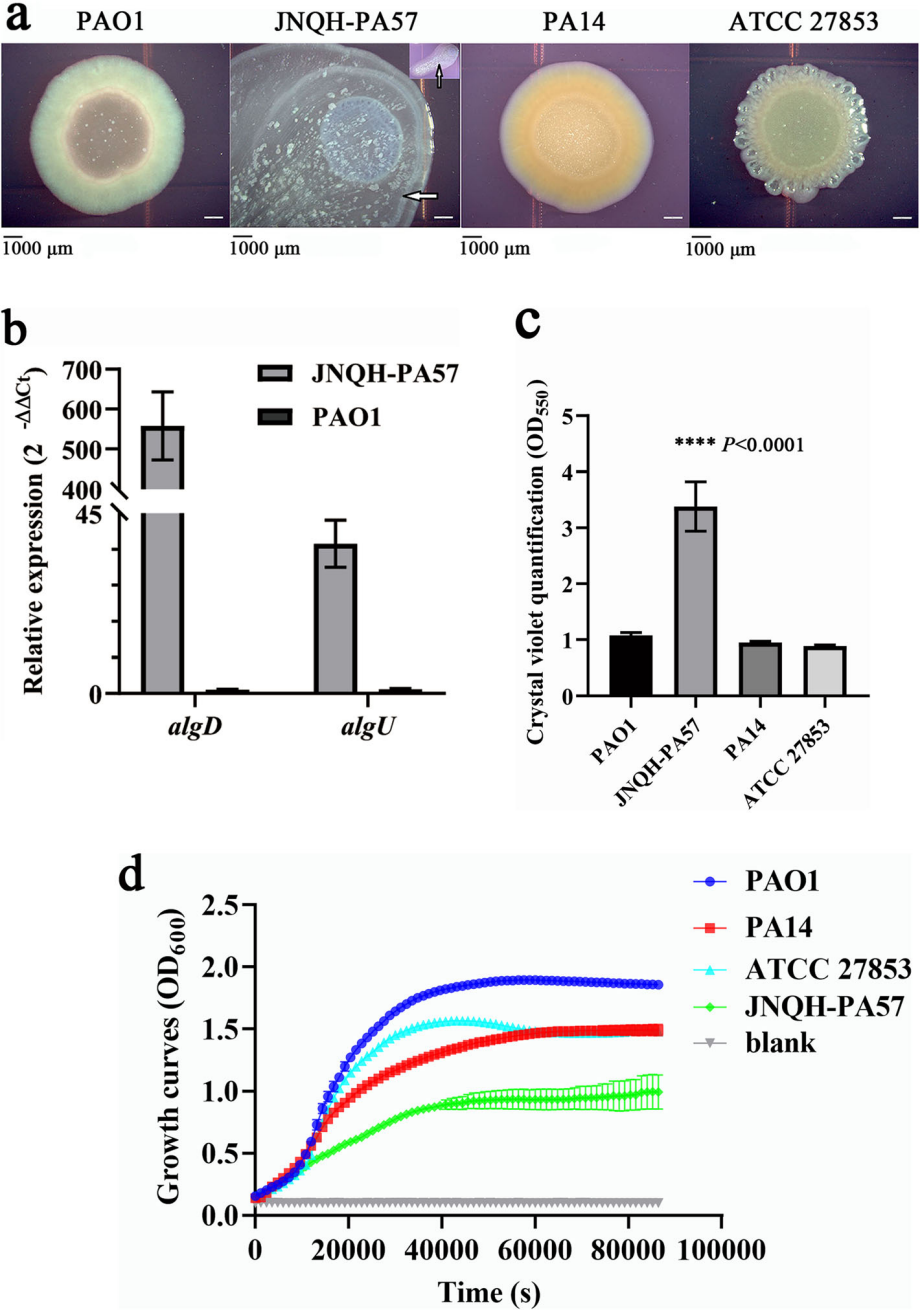
Colony morphology, alginate biosynthesis related genes expression and biofilm formation of *P. aeruginosa* JNQH-PA57. **a** Colony morphology of four *P. aeruginosa* strains cultured at 25 °C on LB agar plates supplemented with Coomassie blue and Congo Red. The white arrow points to the exopolysaccharide produced by JNQH-PA57. **b** Expression of *algD* and *algU* genes in JNQH-PA57 and PAO1 measured by qRT-PCR. Each experiment was done in triplicate. **c** Biofilm formation of four *P. aeruginosa* strains via crystal violet staining. **d** Comparison of the growth of *P. aeruginosa* JNQH-PA57 and three other *P. aeruginosa* reference strains on LB. Representative growth curves of JNQH-PA57, PAO1, PA14 and ATCC 27853 were measured via microplate method

The colony morphology experiment showed that a distinctive mucoid colony was observed in JNQH-PA57 but not in PAO1, PA14 and ATCC 27853, revealing an alginate overproduction from the former strain (Fig. 7a). The expression analysis showed that the transcriptional levels of both *algU* and *algD* in the JNQH-PA57 were significantly higher than those in the nonmucoid reference strain PAO1 (Fig. 7b). The up-regulation in the transcription of the alginate biosynthetic operon might explain mucoid phenotype of this strain. Specifically, the SNP distribution analysis revealed that the presence of a deleted mutation at nt 424 in *P. aeruginosa* JNQH-PA57 *mucA* gene, resulting in a frameshift variant, with the formation of a premature stop codon, likely account for the overproduction of alginate and further leading to the mucoid phenotype of this strain. Biofilm formation and growth curve analysis showed that mucoid strain JNQH-PA57 formed a more robust biofilm whereas exhibited an attenuate planktonic growth compared to PAO1, PA14 and ATCC 27853 (Fig. 7c and d).

### Multiple factors contribute to carbapenem resistance in *P. aeruginosa* JNQH-PA57

Antimicrobial resistance (AMR) genes searching results showed that JNQH-PA57 harbors 47 genes related to antibiotic and disinfectant resistance, most of which (39 genes) are efflux pump-related genes. Six are antibiotic inactivation genes and only two of them are antibiotic target alternation genes (Fig. 3 and Additional file 8: Table S8). Remarkably, JNQH-PA57 possesses 2 chromosomal β-lactamase genes, *bla*_PDC-12_ and *bla*_OXA-488_.

To study whether the carbapenem resistance phenotype of this strain was associated with OprD mutations, nucleotide and amino acid sequences of this porin in JNQH-PA57 were blasted with the OprD in PAO1. Ten non-synonymous mutations were presented in OprD of JNQH-PA57, including T103S, K115T, F170L, E185Q, P186G, V189T, R310E, A315G, P417* (* represents a stop codon) and G425A.

**Fig. 8:**
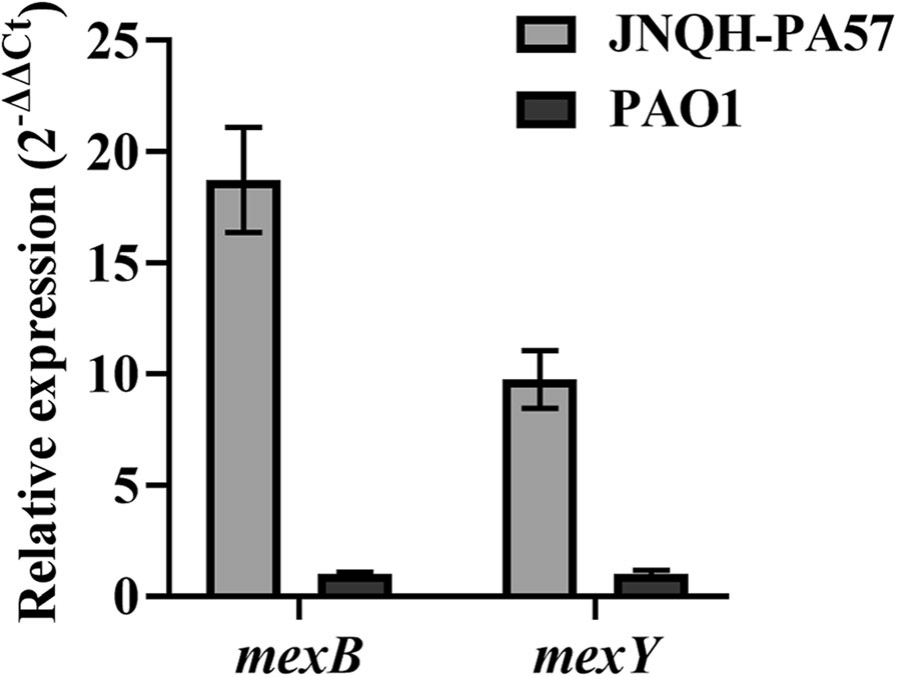
Expression of *mexB* and *mexY* genes in JNQH-PA57 and PAO1 measured by qRT-PCR. Each experiment was done in triplicate

The expressions of two major resistance-nodulation-division (RND) family efflux pumps (MexAB-OprM and MexXY-OprM) were investigated in this study used *mexB* and *mexY* as target genes, respectively. The transcription levels of *mexB* and *mexY* in JNQH-PA57 significantly increased 18.75-and 9.75-fold, compared to those in the reference strain PAO1 (which is sensitive to carbapenem) (Fig. 8).

## Discussion

Having a repertoire of highly flexible genes, the opportunistic pathogen *P. aeruginosa* shows a great deal of diversity thriving in different habitats [31]. The extensive genome plasticity of this pathogen, in large part, is provided by pathogenicity islands [31]. One of the well-characterized pathogenicity islands PAPI-1 is an ICE that encompasses a number of virulence determinants [30]. In addition to the PAPI-1 first described in *P. aeruginosa* PA14, several different ICEs that belonged to PAPI-1 family have been identified in abundant *Pseudomonas* genomes and confer multiple adaptive functions such as antibiotics and heavy metal resistance to the hosts [13, 32]. In the current study, a novel ICE_JNPA57_ was identified in the genome of JNQH-PA57 isolated from a clinical source. ICEs are mobile genetic elements, which harbor specific modular structures encoding the complete conjugation machinery (mainly the T4SS) [33]. A previous study revealed that a cluster of ten genes encoded in PAPI-1 was responsible for the synthesis and assembly of the T4SS [34]. This cluster of 10 genes encoding the T4SS was also found to be embedded in the ICE_JNPA57_. Moreover, this T4SS encoding operon in ICE_JNPA57_ shows highly conservation with (up to 98.5% identity) with that in PAPI-1. The integration and excision of ICE into/out of the host chromosome, which relies on an ICE encoded site-directed integrase, is another defining feature of ICEs [33]. Here, we found that the *attL* and *attR* direct repeats flanked by this ICE_JNPA57_ resemble the conserved integration site in almost all PAPI-1 family ICEs [13]. Besides, the boundary operons organization of ICE_JNPA57_, which starts with a ParA family protein and ends with a putative relaxase (TraI) as well as a site-specific recombinase (Int), are also highly conserved with other PAPI-1 family ICEs [13]. These proteins located at both sides of the ICE are respectively correlated with the mobilization and integration of pathogenicity GIs [35, 36]. All these pieces of evidence mentioned above indicated a close evolutionary relationship between PAPI-1 in PA14 and pKLC102 in clone C strains, revealing that the ICE_JNPA57_ identified in our study should be a member of the PAPI-1 family ICEs.

*P. aeruginosa* JNQH-PA57 studied here is a clinical strain with mucoid phenotype. The conversion of *P. aeruginosa* to mucoid alginate-overproducing form is a critical persistence factor of the inextirpable chronic infection caused by this pathogen [37]. The mucoid switch usually results from bereft regulation of the sigma factor AlgU via inactivating in the anti-sigma factor MucA [37]. MucA is a negative regulator for AlgU (a sigma factor that was required for expression of the key alginate biosynthetic gene *algD*) expression, and responsible for preventing AlgU from binding and activating target promoters. Once MucA is inactivated via nonsense and frameshift mutations, the liberated AlgU will activate the genes related to alginate biosynthesis, such as *algD* (encoding a GDP mannose dehydrogenase in the alginate biosynthesis operon), resulting in constitutive production of alginate [37]. The mutation can occur throughout the *mucA* gene, which, in turn, may result in the generation of MucA proteins of different sizes [38]. Herein, we found that MucA variant in JNQH-PA57 is resulted from an nt 424 deletion mutation, leading to the generation of a protein only containing the N-terminal 147 amino acids of MucA (losing almost 50 amino acids compared with the wild type MucA protein). Yin et al. have reported that mucoid phenotype in clinical isolates can be not only modulated by the size of the MucA protein but also influenced by the genotype of the *algU* in a particular strain [38]. The size of MucA determines the cellular localization of this protein and its ability to repress AlgU, while the genotype of the *algU* determines the activity of AlgU [38]. However, no non-synonymous mutations were detected in JNQH-PA57 *algU* gene. Consequently, these results indicated that the mucoidy in this clinical isolate JNQH-PA57 was likely to be driven by the truncation variant of MucA protein.

Additionally, the alginate overproduction mucoid strain would form a thicker biofilm with large extended mushroom-like microcolonies compared to wild-type strains [39]. It was regarded that this mucoid microcolony mode of growth provides a major benefit to chronic colonization of *P. aeruginosa* in the cystic fibrosis (CF) lung, due to the surrounding overproducing alginate, which would protect the bacteria from the host immune system clearance by restraining phagocytosis, withstanding oxidative burst, interfering opsonization and providing an immunomodulatory role [40, 41]. Moreover, the mushroom-like biofilm had been reported to display an up to 100 to 1000-fold higher tolerance to antibiotics compared with the planktonic bacteria [42]. On the other hand, biofilm growth in cystic fibrosis lungs is associated with the slow growth of *P. aeruginosa* [43]. In our case, we observed that the mucoid JNQH-PA57 formed a more robust biofilm, whereas exhibited an attenuate growth compared with other nonmucoid strains. The increasing doubling time and low bacterial metabolic activity of *P. aeruginosa* in the biofilm-growth mode are partly responsible for the tolerance to some antibiotics [43].

*Pseudomonas*-derived cephalosporinase (PDC) is a class C beta-lactamase, which confers *Pseudomonas* strains resistance to all beta-lactams except carbapenems [44]. The β-lactamase OXA-488 is an OXA-50 family β-lactamase, which can only hydrolyze imipenem at a low level and do not significantly hydrolyze meropenem [45]. Although these two β-lactamase encoding genes, including *bla*_PDC_-12 and *bla*_OXA-488_, were detected in JNQH-PA57, they are unlikely to be the dominant factor that significantly influences the carbapenem resistance phenotype of this strain. Studies have demonstrated that permeability reduction of the outer membrane protein OprD was one of the predominant mechanisms of imipenem resistance in *P. aeruginosa* [46, 47]. OprD-mediated carbapenem resistance can result from the reduced expression of *oprD* or the inactivation of this porin through insertion/deletion mutation and/or a premature stop codon [46, 48]. *P. aeruginosa* clinical strains, which carrying an OprD with polymorphisms, particularly the F170L substitution, were found to rapidly develop carbapenem resistance [48]. The F170L mutation is located on loop3 of *oprD*-encoding porin, which, along with loop2, is related to imipenem binding and passage through the porin [49]. Notably, this F170L polymorphism alone was able to contribute to the carbapenem-resistance phenotype in *P. aeruginosa* [48]. In our study, ten non-synonymous mutations (including but not limited to F170L and a premature stop codon presented at N-terminal 417 amino acid site) were observed in OprD of JNQH-PA57. We inferred that the amino acid substitution of F170L and truncation of OprD due to a G > A base substitution at nt 1251 in *oprD* gene might confer imipenem resistance in JNQH-PA57.

Except for AmpC hyperproduction and OprD inactivation, clinical resistance to carbapenems is regarded to require additional mechanisms, such as RND family efflux pumps (especially MexAB-OprM) overexpression [50, 51]. The RND efflux pump MexAB-OprM, which possesses the broadest substrate profile for multiple classes of antibiotics, promotes the extrusion of various antibiotics [50]. Overexpression of this efflux pump had been frequently detected in a good deal of clinical MDR *P. aeruginosa* isolates [52, 53]. Previous studies indicated that MexAB-OprM is capable of exporting β-lactams including meropenem [54]. However, this efflux pump cannot export imipenem because of the lack of a specific heterocyclic side chain in imipenem that is recognized by MexAB-OprM [53, 54]. On the other hand, it has been reported that overexpression of MexAB-OprM was always combined with the increased expression of MexXY-OprM in the carbapenem-resistant *P. aeruginosa* strains [8]. The isolates with the latter efflux pump profile were resistant or with intermediate resistance to both imipenem and meropenem [8]. We obtained the same results with these previous reports: both MexAB-OprM and MexXY-OprM efflux pumps were overexpressed in this carbapenem-resistant strain JNQH-PA57.

## Conclusion

In summary, several genomic features of *P. aeruginosa* JNQH-PA57 were identified based on the whole-genome sequencing. Phylogenetic analysis showed that JNQH-PA57 has a high genetic relationship with PA14. Comparative genomic analysis revealed that JNQH-PA57 possesses several genomic islands, prophages, as well as a PAPI-1 family ICE, which are absent in the genome of PAO1. JNQH-PA57 presented a distinctive alginate overproduction mucoid phenotype. A deleted mutation at nt 424 resulting in a frameshift variant of *mucA* gene in JNQH-PA57, further led to the upregulated expression of *algU* and *algD*, might account for the mucoid phenotype of this strain. Finally, multiple factors were found to contribute to carbapenem resistance in *P. aeruginosa* JNQH-PA57. It was speculated that the interplay between impaired OprD porin, overexpression of the MexAB-OprM and MexXY-OprM efflux pumps, and chromosomal β-lactamase OXA-488 expression, synergistically with the alginates-overproducing protective biofilm, conferred the high carbapenem resistance to *P. aeruginosa* JNQH-PA57. The dataset presented in this study provided an essential genetic basis for the comprehensive cognition of the physiology, pathogenicity, and antibiotics resistance mechanisms of this multidrug-resistant *P. aeruginosa* mucoid strain.

## Methods

### Ethics

The current study focused only on characterizing an MRD clinical isolate *P. aeruginosa* with mucoid phenotype. This isolate was retrospectively retrieved from the Bacteria Bank hosted in the Department of Laboratory Medicine, the First Affiliated Hospital of Shandong First Medical University Hospital, Jinan. Detail clinical information of the patient was not required in this study. Since the microbial culture had been ordered by physicians because of their necessity for clinical management, the patient’s informed consent was not required and not collected. The Ethics Committee of the First Affiliated Hospital of Shandong First Medical University exempted this study from review and the Review Board also waived the requirement for informed consent.

### Bacterial strain, culture conditions, and antibiotic susceptibility tests

The isolated JNQH-PA57 was grown in Mueller-Hinton agar (MHA) (Oxoid, Hampshire, United Kingdom) at 37 °C for 24 h. The species identification was determined with Microflex LT/SH MALDI-TOF mass spectrometer (Bruker, Germany). The antimicrobial susceptibility of this stain was performed using MIC evaluation via the E-test method for the following antimicrobial agents: piperacillin, piperacillin/tazobactam, ticarcillin/clavulanic acid, ceftazidime, cefepime, aztreonam, imipenem, meropenem, amikacin, tobramycin, levofloxacin, ciprofloxacin, according to the manufacturer’s guidelines (AB Biodisk, Sweden). For colistin, the MIC was determined via a broth microdilution method, according to the Clinical and Laboratory Standards Institute (CLSI) guideline. *P. aeruginosa* ATCC 27853 served as a quality control strain for susceptibility testing. The interpretation of the results was based on the CLSI 2020 guideline [55].

### Phenotypic tests for carbapenemase detection

A phenotypic carbapenemase assay compared carbapenem activity with and without the presence of inhibitors as previously reported [56], with slight modification. Briefly, combined-disc tests of imipenem/meropenem alone and with 400 μg of PBA or 292 μg of EDTA or both of PBA and EDTA were assessed for the identification of the different type of carbapenemase production. PBA was dissolved in DMSO at a concentration of 20 mg/mL. Anhydrous EDTA was dissolved in distilled water at a concentration of 0.1 M. 20 μL PBA solution (containing 400 μg of PBA) and 10 μL EDTA solution (containing 292 μg of EDTA) were dispensed onto commercially available imipenem/meropenem discs respectively or simultaneously. The phenotypic assay was performed by inoculating the bacterial suspension onto the MHA. Then the MHA plates were incubated at 37 °C overnight. The diameter of the growth inhibitory zone around four imipenem/meropenem discs was measured and compared. Enlargement of the inhibition zone by ≥5 mm was considered as a positive combined-disc test result.

### Bacterial DNA extraction, genome sequencing and annotation

A single colony of *P. aeruginosa* JNQH-PA57 grown on Columbia blood plate (Hapo, China) was inoculated into 5 mL of LB medium and shaken at 180 rpm at 37 °C for 18 h. Bacterial cells were collected by centrifugation and the genomic DNA was extracted with the genomic DNA purification kit (Wizard, USA) according to the manufacturer’s instruction, and the DNA integrity was checked on the agarose gel. Next, two independent genomic DNA libraries were prepared for Illumina and Oxford nanopore systems. The combination of long-read Nanopore minION and short-read Illumina NovaSeq 6000 platforms were used to generate the complete genome sequence of *P. aeruginosa* JNQH-PA57. The hybrid genome assembly was performed with unicycler v0.4.8, which allows for both short Illumina reads (accurate) and long Nanopore reads (less accurate) to be used in the conservative mode [57]. The short Illumina reads with high accuracy (Q30 > 85%) were aligned against the long Nanopore reads (as a reference), to correct random sequencing errors and then generate a genome assembly of high accuracy. Then the accurate short Illumina reads were aligned with software Bowtie2 and the final assembly was polished with the Pilon for several rounds to reduce the rate of mismatches and small insertions/deletions [58]. The final genome consensus sequence resulted in one circular replicon of 6,747,067 bp. The complete genome sequence of *P. aeruginosa* JNQH-PA57 was annotated with NCBI Prokaryotic Genome Annotation Pipeline (PGAP), using the method of best-placed reference protein set (GeneMarkS-2+ v4.12) [59]. tRNA genes and rRNA genes were predicted utilizing tRNAscan-SE [60] and RNAmmer [61], respectively. The nucleotide sequence of JNQH-PA57 has been submitted and deposited in the NCBI Nucleotide database (with the accession number of NC_CP060086.1).

### Phylogenetic analyses

To explore the genetic diversity and phylogenetic relationships between JNQH-PA57 and other clinical *P. aeruginosa* strains, SNPs were called from 158 genomes of *P. aeruginosa* strains (including 157 publicly available complete genomes of clinically isolated *P.aeruginosa* strains from the PGDB and JNQH-PA57 from this study) using snippy v. 4.4.1 (https://github.com/tseemann/snippy). Alignments were filtered for recombinations using Gubbins v. 2.4.1 [62] and core SNPs extracted using *SNP-sites* v. 2.5.1 [63]. An approximately-maximum-likelihood phylogenetic tree from alignments of nucleotide sequences was inferred with FastTree [64] and the visualization and annotation of the phylogenetic tree were carried out using an online tool Interactive Tree of Life (iTOL) (https://itol.embl.de/) [65].

### Genomic analysis

Complete genome comparison of JNQH-PA57 with three wildly studied *P. aeruginosa* reference strains was carried out by BLASTn search using BRIG v0.95 [66]. GIs and prophages were labeled in their respective locations. Multiple sequence alignment analysis of *P. aeruginosa* JNQH-PA57 and PAO1, PA14 was performed using progressive Mauve with default setting [67]. GIs in the *P. aeruginosa* JNQH-PA57 genome were predicted with the Island Viewer 4 server [25] based on the prediction method IslandPath-DIMOB [68]. The online software PHASTER was used to predict the prophages in the genome of this strain [26].

COG comparison analysis was performed with VennDiagram in R-platform [69]. Proteins present exclusively in an individual strain and those shared between two or three strains were counted based on Mauve and COG blast analysis, and ultimately represented in Venn diagrams as reported previously [19].

Identification of putative ICE_JNPA57_ in the JNQH-PA57 genome was performed using a web-based tool ICEfinder (https://db-mml.sjtu.edu.cn/ICEfinder/ICEfinder.html) based on an updated database ICEberg 2.0 [28]. Visualizing comparative analysis of ICEs that belongs to PAPI-1 families and gene ortholog prediction was carried out with Easyfig v2.2.3 [70].

A set of genes related to the antibiotic resistance in *P. aeruginosa* JNQH-PA57 were identified using the online CARD (https://card.mcmaster.ca/home) [71]. Identity and coverage thresholds were set to 90%. The sequences of the resistance genes and OprD encoding gene were compared with reference strain PAO1 and those deposited at GenBank using BLASTn (http://www.ncbi.nih.gov/BLAST).

### Growth curve measurement, biofilm detection and colony morphology assay

The growth curves of *P. aeruginosa* strains were measured via a microplate method. Overnight cultures of the strains were adjusted with fresh LB to OD_600_ of 0.01 and then transferred (200 μl) into each well of a 96-well cell culture plate (Corstar, USA) with a cover. Then the microplate was incubated at 37 °C for 24 h with being shaken automatically at one-minute intervals and optical density at 600 nm was determined every 20 min with a microplate reader (Thermo, USA).

Biofilm formation was assayed in 96-well plates with crystal violet staining as described previously [72]. Briefly, overnight cultures were adjusted with fresh LB to the same OD_600_ and then diluted in a fresh LB (1:100). Further, 100 μl of these diluted cultures were transferred into a new 96-well plate and incubated at 37 °C statically for 24 h with lid. Then the liquid cultures were discarded by suction, and the 96-well plate was washed with distilled H_2_O to remove media and unattached cell material. 125 μl 0.1% crystal violet staining solution was added per well and incubated at room temperature for 15 min, followed by removing the staining solution and washing the plate with H_2_O. After the plates were dried at room temperature, 125 μl 30% acetic acid solution was added to each well to destain the pellicle biofilm ring for 15 min at room temperature. The violet acetic acid solution was transferred into a fresh 96-well plate, and the absorbance was measured at 550 nm.

Colony morphology of *P. aeruginosa* was detected by culturing the strains at 25 °C on 1% tryptone agar plates supplemented with 20 μg/mL coomassie blue and 40 μg/mL congo red [73] for 3 days.

### Gene-expression analysis

For the gene-expression assays, the strains were grown overnight, then diluted in a fresh culture (1:100) and grown to the early exponential phase (OD_600_ = 1.0) in LB at 37 °C in a shaking incubator. Total RNA was extracted using TRIpure reagent (Biofit, China) as described in the manual. Total RNA (500 ng) from all isolates was reverse transcribed into single-stranded cDNA using RT6 cDNA Synthesis Kit Ver 2 (TSINGKE, China). QRT-PCR experiments were carried out using 2 × T5 Fast qPCR Mix (SYBR Green I) (TSINGKE, China). Primers specific for the amplification of genes related to alginate biosynthesis (*algU* and *algD*), efflux proteins (*mexB* and *mexY*) were listed in additional file 9 (Table S9). And the amplifications were performed with the LineGene 9600 Plus system (BIOER, China). A ribosomal protein encoding gene, *rpsL*, was used as a reference gene for normalizing the expression levels of target genes [74]. Relative transcript levels were determined by the comparative standard curve method.

## Supplementary Information

**Additional file 1: Table S1.** List of the genomic features of mucoid strain *P. aeruginosa* JNQH-PA57 revealed from the complete genome (this study) and those representative *P. aeruginosa* clinical isolates listed in the *Pseudomonas* Genome Database (PGDB).

**Additional file 2: Table S2.** Strains of clinically isolated *P. aeruginosa* used in this study downloaded from *Pseudomonas* genome database based on the availability of complete genomes

**Additional file 3: Table S3.** Detail information of four large insertion regions which absent in *P. aeruginosa* PAO1 genome but exist in *P. aeruginosa* JNQH-PQ57 genome

**Additional file 4: Table S4.** Detail information of GIs identified in *P. aeruginosa* JNQH-PQ57 genome with IslandPath-DIMOD method

**Additional file 5: Table S5.** Detail information of prophages identified in *P. aeruginosa* JNQH-PQ57 genome

**Additional file 6: Table S6.** Detail information of ICE_JNPA57_

**Additional file 7: Table S7.** COGs uniquely identified in *P. aeruginosa* JNQH-PA057 but absent in PAO1, ATCC 27853 and PA14

**Additional file 8: Table S8.** AMR profile of the *P. aeruginosa* JNQH-PA57

**Additional file 9: Table S9.** Primers used for qRT-PCR (f, forward, sense, and r, reverse, antisense)

## Data Availability

The dataset of complete genome of *P. aeruginosa* JNQH-PA57 sequence has been deposited to National Center Biotechnology Information with the accession number CP060086 (https://www.ncbi.nlm.nih.gov/nuccore/NZ_CP060086.1).

## Abbreviations

MDR: Multidrug-resistant
ICE: Integrative and conjugative element
GI: Genomic island
IS: Insertion sequence
NCBI: National Center for Biotechnology Information
MIC: Minimal inhibitory concentration
PBA: Phenylboronic acid
PGAP: Prokaryotic Genome Annotation Pipeline
CDS: Coding sequences
SNP: Single nucleotide polymorphism
BRIG: BLAST Ring Image Generator
CARD: Comprehensive Antibiotic Resistance Database
ORFs: Open reading frames
T4SS: Type IV secretion system
COG: Clusters of Orthologous Groups
qRT-PCR: Quantitative reverse transcription-PCR
AMR: Antimicrobial resistance
PDC: *Pseudomonas*-derived cephalosporinase
RND: Resistance-nodulation-division
MHA: Mueller-Hinton agar
CLSI: Clinical and Laboratory Standards Institute
iTOL: Interactive Tree of Life
