# Supplementary material for: Comparative genome analysis of multidrug-resistant Pseudomonas aeruginosa JNQH-PA57, a clinically isolated mucoid strain with comprehensive carbapenem resistance mechanisms
Source: BMC Microbiol. 2021 May 1;21:133. doi: 10.1186/s12866-021-02203-4 (PMC8088628; doi:10.1186/s12866-021-02203-4)
Supplement: Supplementary file 1 — Additional file 1: Table S1. List of the genomic features of mucoid strain P. aeruginosa JNQH-PA57 revealed from the complete genome (this study) and those representative P. aeruginosa clinical isolates listed in the Pseudomonas Genome Database (PGDB). [file 12866_2021_2203_MOESM1_ESM.docx]

Table S1 List of the genomic features of mucoid strain *P. aeruginosa* JNQH-PA57 revealed from the complete genome (this study) and those representative *P. aeruginosa* clinical isolates listed in the *Pseudomonas* Genome Database (PGDB).

| Features | JNQH-PA57^a^ | FRD1^a^ | DK1-NH57388A^a^ | PAO1^b^ | PA14 ^b^ | LESB58 ^b^ | PA7 ^b^ | PAK ^b^ | ATCC27853^c^ |
| --- | --- | --- | --- | --- | --- | --- | --- | --- | --- |
| Total length (bp) | 6,747,067 | 6,712,339 | 6,212,531 | 6,264,404 | 6,537,648 | 6,601,757 | 6,588,339 | 6,395,872 | 6,833,187 |
| % GC content | 66.03 | 66.1 | 66.6 | 66.6 | 66.3 | 66.3 | 66.4 | 66.4 | 66.12 |
| Genes (totol) | 6,239 | 6,233 | 5,728 | 5,708 | 6,025 | 6,170 | 6,163 | 5,886 | 6,427 |
| CDS (with protein) | 6,087 | 5,899 | 5,608 | 5,587 | 5,883 | 6,028 | 5,986 | 5,770 | 6,279 |
| rRNA | 12 | 12 | 12 | 12 | 12 | 12 | 12 | 12 | 12 |
| tRNA | 63 | 64 | 63 | 63 | 63 | 67 | 63 | 63 | 66 |
| ncRNA | 4 | 4 | 4 | 44 | 5 | 5 | 4 | 4 | 4 |
| Sequence type | 1197 | 111 | 387 | 549 | 253 | 146 | 1195 | 693 | 155 |
| Accession | NZ_CP060086.1 | NZ_CP010555.1 | NZ_LN870292.1 | NC_002516.2 | NC_008463.1 | NC_011770.1 | NC_009656.1 | NZ_LR657304.1 | NZ_CP011857.1 |

^a^ mucoid phenotype clinical isolates

^b^ popular strains listed in PGDB

^c^ clinical model strain.
