# Supplementary material for: Comparative genome analysis of multidrug-resistant Pseudomonas aeruginosa JNQH-PA57, a clinically isolated mucoid strain with comprehensive carbapenem resistance mechanisms
Source: BMC Microbiol. 2021 May 1;21:133. doi: 10.1186/s12866-021-02203-4 (PMC8088628; doi:10.1186/s12866-021-02203-4)
Supplement: Supplementary file 7 — Additional file 7: Table S7. COGs uniquely identified in P. aeruginosa JNQH-PA057 but absent in PAO1, ATCC 27853 and PA14 [file 12866_2021_2203_MOESM7_ESM.docx]

Table S7 COGs uniquely identified in *P. aeruginosa* JNQH-PA057 but absent in PAO1, ATCC 27853 and PA14.

| COG_ID | Gene location | Gene_locus tag | Annotation |
| --- | --- | --- | --- |
| COG1397 | 1,050,888-1,051,949(-) | H5409_04860 | ADP-ribosylarginine hydrolase Tri1 |
| COG0582 | 1,073,104-1,074,318(-) | H5409_04980 | Prophage integrase IntS |
| COG4977 | 2,614,258-2,615,220(+) | H5409_12310 | HTH-type transcriptional regulator CdhR |
| COG2274 | 2,838,106-2,840,265(-) | H5409_13315 | Toxin RTX-I translocation ATP-binding protein |
| COG0845 | 2,840,275-2,841,501(-) | H5409_13320 | HlyD family efflux transporter periplasmic adaptor subunit |
| COG1696 | 3,575,338-3,576,819(+) | H5409_16350 | Membrane-bound O-acyltransferase family protein |
| COG3772 | 3,597,976-3,598,428(-) | H5409_16455 | Lysozyme RrrD |
| COG0582 | 3,617,004-3,618,221(+) | H5409_16640 | tyrosine-type recombinase/integrase |
| COG3344 | 3,822,076-3,823,404(-) | H5409_17525 | Group II intron reverse transcriptase/maturase |
| COG0603 | 4,283,748-4,284,353(-) | H5409_19700 | 7-cyano-7-deazaguanine synthase |
| COG3639 | 4,300,981-4,301,808(+) | H5409_19765 | Phosphate-import permease protein PhnE |
| COG3038 | 4,785,504-4,786,052(+) | H5409_22110 | Cytochrome b561 |
| COG1487 | 5,441,547-5,441,957(-) | H5409_25175 | tRNA(fMet)-specific endonuclease VapC |
| COG4456 | 5,441,957-5,442,187(-) | H5409_25180 | Antitoxin VapB |
| COG1280 | 5,445,301-5,445,906(-) | H5409_25195 | LysE family translocator |
| COG1566 | 5,482,286-5,483,365(+) | H5409_25375 | Multidrug resistance protein MdtN |
| COG1289 | 5,483,349-5,485,385(+) | H5409_25380 | Fusaric acid resistance protein family protein |
| COG1566 | 5,490,241-5,491,449(-) | H5409_25405 | HlyD family efflux transporter periplasmic adaptor subunit |
| COG1538 | 5,491,439-5,492,896(-) | H5409_25410 | efflux transporter outer membrane subunit |
| COG1335 | 5,493,087-5,493,671(-) | H5409_25415 | isochorismatase family protein |
| COG0625 | 5,494,881-5,495,492(-) | H5409_25425 | Glutathione S-transferase |
| COG0753 | 5,496,763-5,498,211(-) | H5409_25435 | Catalase |
| COG1684 | 6,264,354-6,264,737(-) | H5409_28905 | Flagellar biosynthetic protein FliR |
| COG1987 | 6,264,737-6,265,006(-) | H5409_28910 | Flagellar biosynthetic protein FliQ |
