# Supplementary material for: Comparative genome analysis of multidrug-resistant Pseudomonas aeruginosa JNQH-PA57, a clinically isolated mucoid strain with comprehensive carbapenem resistance mechanisms
Source: BMC Microbiol. 2021 May 1;21:133. doi: 10.1186/s12866-021-02203-4 (PMC8088628; doi:10.1186/s12866-021-02203-4)
Supplement: Supplementary file 8 — Additional file 8: Table S8. AMR profile of the P. aeruginosa JNQH-PA57 [file 12866_2021_2203_MOESM8_ESM.docx]

Table S8 AMR profile of the *P. aeruginosa* JNQH-PA57

| Mechanism | Gene | Gene locus | Product and function |
| --- | --- | --- | --- |
| Antibiotic efflux | *triA* | H5409_00825 | TriA is a membrane protein and required for the triclosan efflux pump function of TriABC-OpmH in *P. aeruginosa*. |
|  | *triB* | H5409_00830 | TriB is a membrane protein and required for the triclosan efflux pump function of TriABC-OpmH in P. aeruginosa. |
|  | *triC* | H5409_00835 | TriC is a resistance nodulation cell division (RND) transporter that is a part of TriABC-OpmH a triclosan-specific efflux protein. |
|  | *mexA* | H5409_02275 | MexA is the membrane fusion protein of the MexAB-OprM multidrug efflux complex. |
|  | *mexB* | H5409_02280 | MexB is the inner membrane multidrug exporter of the efflux complex MexAB-OprM. |
|  | *oprM* | H5409_02285 | OprM is an outer membrane factor protein and is part of the MexAB-OprM, MexVW-OprM, MexXY-OprM and the AmrAB-OprM complex. |
|  | *opmD* | H5409_03885 | OpmD is the outer membrane channel protein of the efflux complex MexGHI-OpmD. |
|  | *mexI* | H5409_03890 | MexI is the inner membrane transporter of the efflux complex MexGHI-OpmD. |
|  | *mexH* | H5409_03895 | MexH is the membrane fusion protein of the efflux complex MexGHI-OpmD. |
|  | *mexG* | H5409_03900 | MexG is a membrane protein required for MexGHI-OpmD efflux activity. |
|  | *bcr-1* | H5409_04220 | Transmembrane protein which expels bicyclomycin from the cell leading to bicyclomycin resistance. |
|  | *armR* | H5409_06800 | ArmR is an antirepressor allosterically inhibits MexR dimer-DNA binding, which is related to up-regulation of MexAB-OprM. |
|  | *mexL* | H5409_07005 | MexL is a specific repressor of mexJK transcription and autoregulates its own expression. |
|  | *mexJ* | H5409_07010 | mexJ is the membrane fusion protein of the MexJK multidrug efflux protein. |
|  | *mexK* | H5409_07015 | mexK is the inner membrane resistance-nodulation-cell division (RND) transporter in the MexJK multidrug efflux protein. |
|  | *mexP* | H5409_07785 | MexP is the membrane fusion protein of the MexPQ-OpmE multidrug efflux complex |
|  | *mexQ* | H5409_07790 | MexQ is the inner membrane transporter of the multidrug efflux pump MexPQ-OpmE. |
|  | *opmE* | H5409_07795 | opmE is an outer membrane factor protein that is part of the multidrug efflux pump MexPQ-OpmE. |
|  | *cpxR* | H5409_09490 | CpxR is a response regulator transcription factor directly involved in activation of expression of RND efflux pump MexAB-OprM in P. aeruginosa. |
|  | *muxA* | H5409_13115 | MuxA is a membrane fusion protein component of the efflux pump system MuxABC-OpmB in P. aeruginosa. |
|  | *muxB* | H5409_13120 | MuxB is one of the two necessary RND components in the P. aeruginosa efflux pump system MuxABC-OpmB. |
|  | *muxC* | H5409_13125 | MuxC is one of the two necessary RND components of the MuxABC-OpmB efflux pumps system in P. aeruginosa. |
|  | *opmB* | H5409_13130 | OpmB is an outer membrane efflux protein cooperation with MuxABC to form the efflux pump system MuxABC-OpmB. |
|  | *oprN* | H5409_13285 | OprN is the outer membrane channel component of the MexEF-OprN multidrug efflux complex. |
|  | *mexF* | H5409_13290 | MexF is the multidrug inner membrane transporter of the MexEF-OprN complex. |
|  | *mexE* | H5409_13295 | MexE is the membrane fusion protein of the MexEF-OprN multidrug efflux complex. |
|  | *soxR* | H5409_14405 | SoxR is a transcriptional activator induces expression of the RND efflux pump-encoding operon mexGHI-opmD. |
|  | *mexX* | H5409_16065 | MexX is the membrane fusion protein of the MexXY-OprM multidrug efflux complex. |
|  | *mexY* | H5409_16070 | MexY is the RND-type membrane protein of the efflux complex MexXY-OprM. |
|  | *mexN* | H5409_19360 | MexN is the inner membrane transporter of the MexMN-OprM multidrug efflux complex. |
|  | *mexM* | H5409_19365 | mexM is the membrane fusion protein of the MexMN-OprM multidrug efflux complex. |
|  | *pmpM* | H5409_19830 | PmpM is a multidrug efflux pump belonging to the MATE family of Pseudomonas aeruginosa. |
|  | *mexV* | H5409_24075 | MexV is the membrane fusion protein of the MexVW-OprM multidrug efflux complex. |
|  | *mexW* | H5409_24080 | MexW is the RND-type membrane protein of the efflux complex MexVW-OprM. |
|  | *oprJ* | H5409_25950 | OprJ is the outer membrane channel component of the MexCD-OprJ multidrug efflux complex. |
|  | *mexD* | H5409_25955 | MexD is the multidrug inner membrane transporter of the MexCD-OprJ complex. |
|  | *mexC* | H5409_25960 | MexC is the membrane fusion protein of the MexCD-OprJ multidrug efflux complex. |
|  | *opmH* | H5409_27985 | OpmH is an outer membrane efflux protein required for triclosan-specific efflux pump function. |
|  | *emrE* | H5409_28070 | EmrE is a small multidrug transporter which confers resistance to tetraphenylphosphonium methyl viologen gentamicin kanamycin and neomycin. |
| Antibiotic inactivation | *bla**_OXA-488_* | H5409_30915 | OXA-488 is an OXA-50 family oxacillin-hydrolyzing class D beta-lactamase |
|  | *aph(3')-IIb* | H5409_04305 | APH(3')-IIb is a chromosomal-encoded aminoglycoside phosphotransferase in P. aeruginosa |
|  | *pdc-12* | H5409_04350 | PDC-12 is an extended-spectrum beta-lactamase found in P. aeruginosa. |
|  | *catB* | H5409_23380 | CatB is a type B chloramphenicol O-acetyltransferase which is responsible for the phenicol resistance. |
|  | *crpP* | H5409_25165 | CrpP is a ciprofloxacin resistance protein and confers resistance to ciprofloxacin by antibiotic inactivation through phosphorylation. |
|  | *fosA* | H5409_21010 | FosA catalyzes the conjugation of glutathione to carbon-1 of fosfomycin rendering it ineffective as an antibacterial drug. |
| Antibiotic target alternation | *arnA* | H5409_07630 | ArnA modifies lipid A and confers resistance to the cationic antimicrobial peptides and antibiotics such as polymyxin. |
|  | *pmrB* | H5409_26975 | PrmB is a histidine protein kinase sensor which modifies Lipid A and is involved in polymyxin resistance |
