# Supplementary material for: Comparative genome analysis of multidrug-resistant Pseudomonas aeruginosa JNQH-PA57, a clinically isolated mucoid strain with comprehensive carbapenem resistance mechanisms
Source: BMC Microbiol. 2021 May 1;21:133. doi: 10.1186/s12866-021-02203-4 (PMC8088628; doi:10.1186/s12866-021-02203-4)
Supplement: Supplementary file 9 — Additional file 9: Table S9. Primers used for qRT-PCR (f, forward, sense, and r, reverse, antisense) [file 12866_2021_2203_MOESM9_ESM.docx]

| Primer | Sequence |
| --- | --- |
| RT-algD.f | CGAGAAGTCCGAACGCCACA |
| RT-algD.r | CATCGGCGGGAAGTCGTAGTC |
| RT-algU.f | CGATGTGACCGCAGAGGATG |
| RT-algU.r | ACTGCTGGATGGTCTGGTGC |
| RT-mexB.f | CATCGTGGAGTTCGCCAAGGAG |
| RT-mexB.r | ACGCCGGTACCGATCGCAT |
| RT-mexY.f | TCGCTGACCCTGACCTTCCG |
| RT-mexY.r | ATGCTGTCCGCCGCCTTCTC |
| RT-rpsL.f | AGGTCACAACCTGCAAGAGC |
| RT-rpsL.r | CTTCGAACGACCCTGCTTAC |

Table S9 Primers used for qRT-PCR (f, forward, sense, and r, reverse, antisense)
